# Maresin-1 and Inflammatory Disease

**DOI:** 10.3390/ijms23031367

**Published:** 2022-01-25

**Authors:** Natsuko Saito-Sasaki, Yu Sawada, Motonobu Nakamura

**Affiliations:** Department of Dermatology, University of Occupational and Environmental Health, 1-1, Iseigaoka, Yahatanishi-Ku, Kitakyushu 807-8555, Fukuoka, Japan; motonaka@med.uoeh-u.ac.jp

**Keywords:** ω-3 polyunsaturated fatty acids, maresin-1, inflammatory diseases

## Abstract

Inflammation is an essential action to protect the host human body from external, harmful antigens and microorganisms. However, an excessive inflammation reaction sometimes exceeds tissue damage and can disrupt organ functions. Therefore, anti-inflammatory action and resolution mechanisms need to be clarified. Dietary foods are an essential daily lifestyle that influences various human physiological processes and pathological conditions. Especially, omega-3 fatty acids in the diet ameliorate chronic inflammatory skin diseases. Recent studies have identified that omega-3 fatty acid derivatives, such as the resolvin series, showed strong anti-inflammatory actions in various inflammatory diseases. Maresin-1 is a derivative of one of the representative omega-3 fatty acids, i.e., docosahexaenoic acid (DHA), and has shown beneficial action in inflammatory disease models. In this review, we summarize the detailed actions of maresin-1 in immune cells and inflammatory diseases.

## 1. Introduction

The human body is exposed to various environmental stimuli which drive appropriate host defense reactions to protect against harmful antigens and/or microorganisms [1]. On the one hand, inflammatory responses are essential to drive host defense actions and the remodeling of organ tissues [2]. On the other hand, an inflammatory reaction sometimes exacerbates the inflammatory response, leading to tissue damage and the development of systemic inflammatory diseases [3]. Because inflammation has been implicated in many human diseases, anti-inflammatory agents should be applied to various inflammatory diseases. Steroid or non-steroidal anti-inflammatory drugs (NSAIDs) are currently available medications for the treatment of these inflammatory diseases; however, these medications have disadvantages due to the fact adverse reactions have been associated with them. Therefore, there is a need to develop some safe agents for the treatment of inflammatory diseases.

The daily diet is one of the essential daily lifestyles to sustain the lives of human beings, in which nutrition comprises their body elements and cell components. Among the various nutritional factors, fatty acids are components of cell membranes that regulate cellular signal transduction [4,5]. Recently, research has increased our knowledge of the beneficial actions of fatty acids on human health [6]. Omega-3 fatty acids are abundant in fish oil and are known to have potential benefits in various inflammatory diseases including asthma, psoriasis, inflammatory bowel disease, and rheumatoid arthritis [7]. Furthermore, lipid mediator metabolites derived from omega-3 fatty acids act on various physiological processes or pathological conditions by regulating lipid metabolism, cell signaling, and inflammation [8,9,10,11].

Maresin-1 is one of the highlighted metabolites from eicosapentaenoic acid (EPA) that shows various strong anti-inflammatory actions in inflammatory diseases. In this review, we summarize the actual impacts of maresin-1 on various diseases and discuss the detailed molecular mechanisms of maresin-1 focusing on its anti-inflammatory action.

### 1.1. The Anti-Inflammatory Actions of Maresin-1

Maresin-1 was first identified in human macrophages and was shown to be a lipid mediator that was active in the process of inflammatory resolution. Studies have shown that human macrophages produce maresin-1 mediated by 14-lipoxylation of DHA and enzymatic hydrolysis from 13S,14S-epoxymaresin [12,13]. Regarding its anti-inflammatory action, maresin-1 has been shown to suppress neutrophil migration [14] and cytokine production by activating CD8^+^ T cells, CD4^+^ T helper (Th1) cells, and Th17 cells. Maresin-1 has also been shown to negatively regulate the transcription factors T-bet and Rorc, prevent Th1 and Th17 differentiation, and simultaneously enhance Foxp3^+^ regulatory T (Treg) cells generation mediated by the GPR32 receptor [15]. 

Considering the action mechanism of maresin-1, a recent study identified one receptor, i.e., a leucine-rich repeat-containing G-protein-coupled receptor 6 (LGR6), which had the structure of GPCRs and was widely identified in various tissues [16]. Another receptor, i.e., retinoic acid-related orphan receptor α (RORα), has been located in the nucleus [17]. Although these two receptors play roles in the action mechanism of maresin-1, the total detailed molecular mechanisms of the resolution of inflammation, host defense, tissue homeostasis, and wound healing are still unclear.

**Figure 1:**
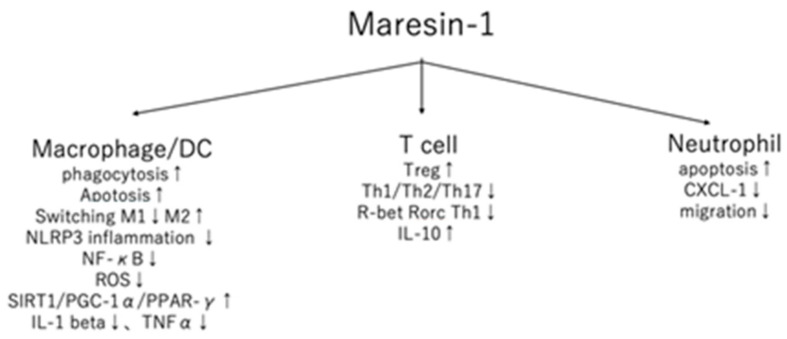
The detailed action of maresin-1 in immune cells. ↑ upregulated, ↓ downregulated.

Maresin-1 has been demonstrated to have various actions on immune cells. In this section, we review the influence of maresin-1 on dendritic cells (DCs)/macrophages, T cells/regulatory T cells, and neutrophils, as shown in Table 1 and Figure 1.

### 1.2. DCs/Macrophages

External pathogens and apoptotic cells exacerbate tissue inflammation. However, DCs and macrophages dampen these inflammatory responses by phagocytosis of these pathogens and apoptotic cells for inflammatory resolution. Maresin-1 has been shown to enhance phagocytotic activity in macrophages and promote anti-inflammatory action [18].

Maresin-1 has been shown to promote polarization of CD11c^−^CD206^+^ (M2) macrophages and inhibit polarization of CD11c^+^CD206^−^ (M1) macrophages [19]. The M2 macrophages secreted anti-inflammatory cytokines such as IL-10 and TGF-β, which accelerated tissue remodeling and the clearance of apoptotic debris by phagocytosis [20]. This action of maresin-1 negatively regulated the inflammatory response.

In addition, maresin-1 has been shown to impair inflammatory cytokine production in anti-inflammatory actions. Maresin-1 suppressed the production of IL-1β [21] and TNF-α [22]. In addition, maresin-1 suppressed the production of TNF-α and IL-6 through the suppression of the SIRT1/PGC-1α/PPARγ pathway [23].

### 1.3. T Cells

Studies have demonstrated that maresin-1 has anti-inflammatory effects on T cells. Maresin-1 suppressed the induction of CD4+ cells, CD8+ cells, and Th17 cells by downregulation of T-bet and Rorc expression [15]. On the contrary, maresin-1 enhanced the induction of Tregs and the production of anti-inflammatory cytokine IL-10. Maresin-1 negatively regulated IL-23 receptor expression on γδ T cells through downregulation of RORγ and internalization of IL-23 receptor [24]. Therefore, maresin-1 suppressed induction of effector cells and induced Treg expansion and anti-inflammatory cytokine production in T cells [15].

### 1.4. Neutrophils

Neutrophils are involved in the innate immune response and rapidly migrate to infection or injury sites to eliminate invading microorganisms by phagocytotic activity. Maresin-1 has been shown to suppress neutrophil infiltration and decrease the production of CXCL1, which is one of the major chemokines to recruit neutrophils. Apoptosis is a programmed cell death process that prevents the release of cytotoxic contents in cells, and neutrophils contain various abundant cytotoxic substances. Maresin-1 has also been shown to promote apoptosis of neutrophils to induce the resolution of inflammatory response [25].

## 2. The Inflammatory Actions of Maresin-1 in Inflammatory Diseases

In this section, we introduce the detailed action of maresin-1 in various diseases, especially, neurological disorders, pain, respiratory diseases, diabetes and obesity, kidney disease, liver disease, arthritis, colitis, infectious disease, cardiovascular diseases, and cutaneous diseases.

### 2.1. Neurology

Neurological actions are essential for human beings to sustain life and the dysregulation of neurological action leads to various severe disorders, such as Alzheimer’s disease. Because there is no radical treatment for these neurological diseases, novel therapeutic or preventive options are desired. Consistently, several studies have identified that maresin-1 had beneficial effects against these neurological diseases. It has been shown, in mouse models, that maresin-1 showed an inhibitory effect on acute neurological damage such as spinal cord injury and cerebral ischemia. In addition, maresin-1 has been shown to impair the disease progress of a chronic degenerative disease, Alzheimer’s disease, by the action mechanism of an inflammatory resolution against the deposition of amyloid-β protein-mediated inflammation [26].

A spinal cord injury accelerates the inflammatory response in neurological tissue and causes delayed remodeling of neurological function. Therefore, early resolution of inflammation associated with a spinal cord injury is required to avoid an excessive inflammatory response in the inflammatory site. Maresin-1 has been shown to be active in the resolution of inflammatory after a spinal cord injury. In an animal model of spinal cord injury, maresin-1 accelerated the resolution of neutrophils and decreased macrophage infiltration at the lesion, which contributed to neurological recovery after a spinal cord injury [27].

In an animal model of brain infarction, maresin-1 also impaired inflammatory reactions in lesions and reduced neurological defects [28]. Epigenetic modification is a powerful gene regulatory mechanism through DNA and/or DNA-binding proteins, such as histone, which modulate open chromatin sites to enhance transcriptional activation [29,30]. Interestingly, maresin-1 has been shown to activate epigenetic modification mediated by silent information regulator 1 (SIRT1 signaling), which is one of the histone deacetylases, to negatively regulate acetylation of nuclear factor kappa B and Bax expression, as well as to reduce downstream proinflammatory cytokines, such as TNF-α and IL-1, leading to a reduction in infarction size and the neurological defects after cerebral ischemia/reperfusion [31].

Epidemiological studies have shown the benefits of DHA intake to reduce the risk of Alzheimer’s disease [32]. Furthermore, oral intake of DHA has been shown to impair disease activity in an animal model of Alzheimer’s disease [33]. These findings suggest that maresin-1, which is a derivative of DHA, should show therapeutic efficacy against Alzheimer’s disease. Consistently, maresin-1 has also shown potential benefits associated with neurological degenerative diseases. For instance, Alzheimer’s disease is a representative neurological degenerative disease, and its incidence is increasing worldwide. Because there are few therapeutic options that have obtained a satisfactory level of clinical use, various research approaches are currently being conducted. In an animal model of Alzheimer’s disease, amyloid-β42 protein was believed to play a central role in the pathogenesis of Alzheimer’s disease by causing an inflammatory response in the amyloid-β42 proteins deposited in the brain. Maresin-1 has been shown to decrease the production of proinflammatory cytokines, such as TNF-α and IL-6, while increasing the secretion of the anti-inflammatory cytokines, IL-2 and IL-10, by the regulation of the amyloid-β42 protein [34,35,36].

### 2.2. Pain

The therapeutic potential of maresin-1 against pain has been investigated in various animal models. Dorsal root ganglion (DRG) neurons were used to cause neurological pain by capsaicin and vincristine sulfate injection into the hind paw plantar surface, and these stimuli enhanced pains mediated by the transient receptor potential V1 (TRPV1), which was suppressed by maresin-1 treatment [37]. Consistently, maresin-1 inhibited the TRPV1 agonist-induced activation of pain [38].

The beneficial effect of maresin-1 has also been shown in allodynia and thermal hyperalgesia, which relate to nerve hypersensitivity to pain [39]. Maresin-1 has also been shown to be effective in chronic pain, when administered between the L4 and L6 vertebrae of the spinal cord and the analgesic effect was observed for 5 days [40]. Regarding the mechanisms, maresin-1 decreased proinflammatory cytokines (IL-1β, IL-18, and TNFα) and reduced NLRP3 inflammasome, leading to the impairment of cell death and positive activation of NF-κB/p65-mediated inflammation and pain [41]; the mechanical and thermal hypersensitivity enhanced the IL-1β and IL-18 levels and the expression of NLRP3 inflammasome components, which were markedly suppressed by maresin-1 treatment [42].

Polyunsaturated linoleic acid decreases in Ca^2+^ ions in the cytosol of neurons and astrocytes in an ischemia model [43]. Because maresin-1 is a derivative of polyunsaturated linoleic, maresin-1 might also show therapeutic potential against brain stroke as another possible action mechanism.

### 2.3. Respiratory Diseases

The anti-inflammatory effects of maresin-1 have been confirmed in various respiratory diseases. Organic dust becomes the cause of chronic airway inflammation, especially obstructive pulmonary disease, due to increased neutrophil infiltration and the production of TNF-α and IL-6. Maresin-1 suppressed proinflammatory cytokine production and intracellular adhesion molecule-1 (ICAM-1) expression in bronchial epithelial cells under organic dust exposure [44]. Maresin-1 significantly decreased bronchoalveolar lavage neutrophil infiltration, and the secretion levels of IL-6, TNF-α, and chemokine C-X-C motif ligand 1 (CXCL1) [45].

High-dose maresin-1 treatment has been shown to impair lung inflammation in an LPS-induced acute lung injury (ALI) mouse model [46]. Infiltrating neutrophils enhance the inflammatory response and release proteolytic enzymes and reactive oxygen species to cause excessive tissue damages. Neutrophil apoptosis was accelerated under LPS-induced ALI, which was impaired by maresin-1 due to the enhancement of caspase-dependent neutrophil apoptosis [25].

Pulmonary fibrosis is a progressive, chronic lung epithelial injury that results in an uncontrolled fibrotic response. Epithelial-to-mesenchymal transition (EMT) is thought to have a pathogenetic role in pulmonary fibrosis by causing epithelial cells to irreversibly shift to a mesenchymal phenotype. TGF-β1 plays a role as a positive driver for EMT in pulmonary fibrosis and enhances collagen synthesis and fibroblast proliferation. The concentration of TGF-β1 in bronchoalveolar lavage fluid and fibrosis markers, such as fibronectin and α-SMA, were suppressed by maresin-1 administration [47]. Maresin-1 has also been shown to inhibit proliferation, migration, and differentiation in fibroblast by suppressing phosphorylation of decapentaplegic homolog 2/3 (Smad2/3) and extracellular-signal-related kinase 1 and 2 (ERK1/2) in a dependent manner [48].

Bronchial asthma is a chronic inflammatory disease of the lower respiratory tract mediated by Th2 allergic reactions, which are essential to exacerbate an inflammatory response by NF-κB-induced ICAM-1 expression in vascular endothelial cells and lung epithelial cells to increase eosinophil adhesion to endothelial cells and to promote Th2 differentiation. Epidemiological studies have suggested that increased dietary intake of fish oil containing omega-3 fatty acids was associated with a reduced risk of asthma [49], suggesting a possible therapeutic efficacy of maresin-1 for asthma. In a mouse model of OVA-induced asthma, maresin-1 markedly suppressed activation of the NF-κB signaling pathway and its downstream cascades, such as COX-2 and ICAM-1 [50]. Maresin-1 enhanced the suppression of innate lymphoid cell type 2 (ILC2) in a TGF-β-dependent manner [51].

Pulmonary ischemia/reperfusion injury causes obstructive bronchiolitis, such as pulmonary thrombolysis and oxidative stress generation plays some part in its pathogenesis. A study has shown that maresin-1 impaired oxidative and antioxidant production leading to protection of the lung tissues [52].

### 2.4. Diabetes and Obesity

The role of inflammation in the development of type 2 diabetes mellitus and its complications has received increasing attention because the incidence is currently increasing in the world. IL-1β, IL-6, and CRP are considered to be prognostic factors in diabetes [53], and anti-inflammatory lipid mediators such as maresin-1 should prevent the worsening of diabetic retinopathy by converging such adipose inflammation and altering insulin resistance and adipokine secretion [54]. Diet-induced obese mice treated with maresin-1 also exhibited decreased proinflammatory cytokines such as TNF-α and IL-1β [55]. Furthermore, high-fat diet-induced hyperglycemia has been improved by maresin-1 [56] and maresin-1 has enhanced the repair function of macrophages and has also promoted the diabetic wound repair ability [57].

Nonalcoholic fatty liver disease (NAFLD) associated with obesity is a pathological condition caused by endoplasmic reticulum stress and activation of unfolded protein responses. Maresin-1 has been shown to inhibit the endoplasmic reticulum stress of hepatocytes and to enhance the phagocytic activity of Kupffer cells, leading to the protection of hepatocytes from apoptosis [58]. DHA supplementation has been shown to impair metabolic abnormalities in children with NAFLD [59], suggesting a possible therapeutic potential of maresin-1 against NAFLD. Studies have shown that maresin-1 ameliorated hepatic steatosis by inhibiting AMPK/SERCA2b-mediated endoplasmic reticulum stress [60]; impaired hepatic lipidosis by inhibiting AMPK activation and inducing autophagy [61]; and suppressed liver injury by increasing the expression and transcriptional activity of RORα [62].

### 2.5. Kidney Disease

Leukocyte-mediated inflammation also plays a pathogenetic role in acute kidney injury. One study reported that maresin-1 influenced the survival of neutrophils and subsequently prevented kidney injury. The macrophages produced 14S,21R-dihydroxydocosahexaenoic acid (14S,21R-diHDHA), which repaired the vascular endothelium and contributed to the protection of kidney function [63].

Inflammation and fibrosis are also important pathologies in diabetic nephropathy. In a mouse model of diabetic nephropathy, maresin-1 exerted a protective effect on glomerular mesangial cells by decreasing the expression of ROS, NLPR3, caspase-1, and IL-1β, which are responsible for the development of diabetic nephropathy [64].

Inflammation and oxidative stresses are also involved in ischemia/reperfusion-induced renal injury; TLR4-mediated inflammatory response and the signal pathway mediated by ERK, JNK, and P38 MAPK play an important role. Maresin-1 treatment has been shown to decrease the expression levels of ERK, JNK, and P38 MAPK [65].

### 2.6. Liver

The effects of maresin-1 in hepatic injury have also been investigated in a mouse model of acute hepatic injury. Maresin-1 inhibited reactive oxygen species and inflammatory cytokines and chemokines, such as IL-6, IL-1β, and monocyte chemotaxis protein-1 (MCP-1), and suppressed carbon tetrachloride-induced liver injury [66]. In another acute liver injury mouse model induced by concanavalin A, maresin-1 also impaired liver injury by reducing hepatocytes apoptosis while increasing apoptosis of mouse macrophages, in addition to reducing ROS in macrophages [67].

Hepatic ischemia-reperfusion injury causes liver dysfunction after liver surgery. In a rat model, maresin-1 impaired hepatic injury by activating hepatocyte and promoting nuclear localization of Nrf-2, leading to a decrease in NF-κB activity [68].

In another study, the hepatoprotective effect of maresin-1 was abrogated by pretreatment with Boc2 (lipoxin A4 receptor antagonist), and the hepatoprotective effect of maresin-1 was further reversed by inhibition of Akt. Thus, maresin-1 protected the liver from hepatic ischemia-reperfusion injury mediated by the ALXR/Akt signaling pathway [69].

### 2.7. Arthritis

Several studies have shown the therapeutic potential of maresin-1 against arthritis. The concentration of maresin-1 in synovial fluid of rheumatoid arthritis patients has been shown to be related to disease activity [70], suggesting that maresin-1 might have a protective role in the development of rheumatoid arthritis. The serum level of maresin-1 was also lower in inactive rheumatoid arthritis than active rheumatoid arthritis. An inverse correlation was observed between the FoxP3/RORc ratio and the disease activity score 28, which is a measure of disease activity in rheumatoid arthritis. Furthermore, maresin-1 has been shown to suppress inflammatory response, in a rheumatoid arthritis animal model [71]. Maresin-1 also showed therapeutic potential by elevating intra-articular lavage fluid in a treadmill-loaded mouse model of osteoarthritis. Maresin-1 treatment has been reported to enhance type II collagen in cartilage and to decrease MMP13 in the synovium, mediated by the PI3k/Akt and NF-κB p65 pathways [72].

### 2.8. Colitis

The beneficial effects of EPA and DHA on inflammatory bowel disease have been reported. In a colitis animal model induced by dextran sulfate sodium (DSS) and 2,4,6-trinitrobenzene sulfonic acid, maresin-1 suppressed disease activity in colitis and improved weight loss by decreasing IL-1β, TNF-α, IL-6, and IFN-γ in the acute phase. Maresin-1 also decreased neutrophil migration and ROS production mediated by the NF-κB pathway [73]. Maresin-1 negatively regulated the toll-like receptor 4 (TLR4)-mediated NF-κB pathway [74].

### 2.9. Infectious Diseases

Acute inflammation induced by infections can cause excessive tissue damage, and therefore, the resolution of an acute inflammatory response during the early phase is important for infectious disease regulation. Maresin-1 has also been shown to regulate the inflammatory immune response during an *Escherichia coli* infection [75]. Furthermore, in a sepsis animal model induced by intestinal ligation and puncture, maresin-1 decreased the serum concentration of LPS, promoted bacterial clearance, and protected critical organ functions in addition to improved survival [76]. Furthermore, mitochondrial dysfunction leads to increased ROS production in sepsis, and maresin-1 has been reported to increase mitochondrial membrane integrity by retaining adenosine triphosphate content and decreasing ROS production [77].

During severe sepsis, acute kidney injury is the most severe complication, which has been shown to be impaired by maresin-1 treatment. Neutrophil infiltration is inhibited by maresin-1 via the NF-κB/STAT3/MAPK pathway and negatively modulated proinflammatory cytokine levels [78]. Maresin-1 can also impair myocardial infarction during sepsis and reduce the levels of LDH and CK, leading to the improvement of cardiac function through promoting M2 macrophages differentiation [79].

### 2.10. Cardiovascular Diseases

Cerebrovascular diseases are life-threatening diseases with inflammation responses that can result in vascular damage. Maresin-1 treatment could impair these responses through anti-inflammatory effects in vascular endothelial cells. Maresin-1 has been shown to inhibit TNF-α-induced monocyte adhesion and ROS generation in vascular endothelial cells and smooth muscle cells by causing upregulation of cAMP and downregulation of the transcription factor NF-κβ [80]. Vascular injury activates remodeling of vascular endothelial cells by inflammation and sometimes causes neointima formation leading to re-occlusion in blood vessels. Systemic administration of maresin-1 has been shown to reduce neointima formation, and therefore, should suppress the re-occlusion of blood vessels [81].

There is agreement that chronic inflammation is one of the causes of atherosclerosis. Profiling of the aortic qualities of mediators in Apoe-deficient mice fed a high-fat diet showed increased inflammatory lipid mediators leukotriene B4 and prostaglandin E2, and decreased omega-3 polyunsaturated fatty acid lipid mediators, such as resolvin D2 (RvD2) and maresin-1. Maresin-1 inhibited the progression of atherosclerosis by suppressing necrosis in the atherosclerosis site and macrophage accumulation, and increasing the fibrous coat thickness of smooth muscle cells [82].

Smooth muscle cell-specific TGF-β2 receptor-deficient mice were used to induce localized abdominal aortic aneurysms to confirm the therapeutic potential of maresin-1; maresin-1 treatment suppressed the growth of aortic aneurysms mediated by LGR6 receptor signaling which was responsible for TGF-β2 and MMP2 activity in macrophage-apoptotic smooth muscle cell crosstalk [83].

### 2.11. Cutaneous Diseases

Psoriasis is an inflammatory skin disease that is characterized by scaly erythematous plaques, which can also drive the inflammatory immune reaction to systemic organs. Patients with psoriasis have been shown to have a lower amount of omega-3 fatty acids as compared with healthy controls [84], indicating the importance of a diet with regular intake of foods that provide omega-3 fatty acids. A study reported that consistent fish oil supplement intake impaired psoriatic skin inflammation in approximately 80% of patients with psoriasis [85]. There are several benefits of omega-3 polyunsaturated fatty acid metabolites in inflammatory skin diseases. Maresin-1 has also been known to have anti-inflammatory action in psoriasis [24]. A topical application of maresin-1 showed anti-inflammatory effects in a mouse model of psoriasis induced by imiquimod. Maresin-1 inhibited the production of IL-17A by γδTCR^mid+^ and CD4^+^ cells in the skin by downmodulation of IL-23 receptor (IL-23R) expression in clathrin-dependent internalization mechanisms in γδTCR^mid+^ and CD4^+^ cells [24]. Therefore, topical maresin-1 could become a therapeutic option for the treatment of IL-17-mediated other inflammatory diseases.

There are various types of skin inflammations. For instance, Th1 is involved in the pathogenesis of contact dermatitis and Th2 mediates atopic dermatitis. The pathogenesis of alopecia areata is associated with changes in Th1, Th2, and Th17. Therefore, maresin-1 should show anti-inflammatory actions in various skin diseases.

## 3. Conclusions

In this review, we summarized the therapeutic potential of maresin-1 in various inflammatory diseases. Maresin-1 could be an alternative therapeutic option to overcome the disadvantages of current anti-inflammatory agents. Furthermore, by discovering the effects on other types of cells, such as keratinocytes, the actions of maresin-1 could be clarified. Because the incidence of inflammatory diseases is currently increasing, future basic research and clinical trials for maresin-1 should provide beneficial information on its use for the daily clinical treatment of inflammatory diseases.

## Figures and Tables

**Table 1:** The detailed action of maresin-1 in immune cells. ↑ upregulated, ↓ downregulated.

Macrophage DC	Phagocytosis ↑ M2 Polarization IL-1β ↓, TNF-α ↓, IL-6 ↓, ROS ↓
T cells	Th1, Th2, Th17 induction ↓ Treg ↑
Neutrophil	Apoptosis ↑

## Data Availability

Not applicable.
